# Poor serum uric acid control increases risk for developing hypertension: a retrospective cohort study in China

**DOI:** 10.3389/fendo.2024.1343998

**Published:** 2024-01-31

**Authors:** Zeyin Lin, Shaoyan Wu, Zhe Chen, Weijian Luo, Zhihui Lin, Honghui Su, Dongming Guo

**Affiliations:** ^1^ Department of Ultrasound, the First Affiliated Hospital of Shantou University Medical College, Shantou, China; ^2^ Department of Interventional Ultrasound, the Second Affiliated Hospital of Shantou University Medical College, Shantou, China

**Keywords:** uric acid, hypertension, blood pressure, risk factors, epidemiology

## Abstract

**Background:**

Serum uric acid (SUA) has been suggested as a contributor of hypertension. However, reports on the relationship between changes in SUA and hypertension are limited. Hence, we aimed to investigate the potential impact of SUA, especially its change over time, on hypertension incidence.

**Methods:**

This dynamic cohort included 6052 participants without hypertension at baseline. Participants were categorized into six grades based on whether baseline SUA was high and whether changes in SUA progressed to hyperuricemia or decreased to normal levels. Grades 1 to 6 represented the participants’ SUA control from best to worst. Logistic regression and restricted cubic spline (RCS) models were used to explore the association of the grades of SUA control and hypertension incidence.

**Results:**

During a median follow-up of 6 years, 2550 (42.1%) participants developed hypertension. After adjusting confounding factors, compared to grade 1 with the best control of SUA, the odds ratios for grades 2 to 6 with worse control were 1.347 (1.109-1.636), 1.138 (0.764-1.693), 1.552 (1.245-1.934), 1.765 (1.170-2.663), and 2.165 (1.566-2.993), respectively. RCS indicated a linear correlation between the risk of hypertension and changes in SUA, and an elevated risk in participants with baseline hyperuricemia. Subgroup analyses showed that grades of SUA control had an interaction with systolic (*P* = 0.003) and diastolic blood pressure (*P* < 0.001). Sensitivity analyses further determined the robustness of the result that participants with poor SUA control have a higher risk of developing hypertension.

**Conclusion:**

Poor SUA control, an increase in SUA over time, rises the risk of developing hypertension regardless of whether the initial SUA is normal or not. Initial hyperuricemia will exacerbate this risk. Effective SUA control should be an important measure for primary prevention of hypertension.

## 1 Introduction

Hypertension has been recognized as a serious global public health problem, posing a considerable burden due to its widespread prevalence. An estimated 31.1% of adults (1.39 billion) worldwide had hypertension in 2010 (1). In China, among adults aged 35-75 year, nearly half have hypertension, less than a third are being treated, and fewer than one-twelfth are in control of their blood pressure (2). Hypertension is the leading preventable contributor to premature death and disability worldwide (3). It has been proved that hypertension is the major cause of high morbidity and mortality in cardiovascular diseases and stroke (4, 5). Additionally, hypertension, being a common chronic disease, negatively impacts the quality of life of hypertensive individuals compared to those with normal blood pressure (6). Despite the increasing prevalence and influence of hypertension has become a global challenge, hypertension is controllable and preventable. Timely intervention is an essential measure to reduce the incidence of hypertension and related events. Thus, it is important to identify the risk factors for development of hypertension.

Uric acid is the end product of purine metabolism generated during breakdown of nucleic acids and adenosine triphosphate, and also can be generated from degradation of a purine-rich dietary (7). In humans, due to the lack of urate oxidase, serum uric acid (SUA) can not be further degraded to allantoin, which is excreted freely in the urine (8). When the production of uric acid surpasses kidney excretion, the level of SUA will elevated, causing hyperuricemia (HUA). Growing evidence shows that HUA increases the risk of hypertension incidence (9). Several previous studies (10–12) have suggested that SUA is an independent risk factor for the onset and progression of hypertension. However, most of the relevant researches are cross-sectional studies and focus on identifying the association between baseline SUA and hypertension. Few studies explore the role of changes over time in SUA in the development of hypertension. Baseline SUA reflects the uric acid level at a specific point in time, but SUA levels are dynamic. Uncontrolled SUA levels are likely to further increase the risk of hypertension. Therefore, we conducted this study to investigate the potential impact of SUA, especially its change over time, on future hypertension incidence.

## 2 Methods

### 2.1 Study population

**Figure 1:**
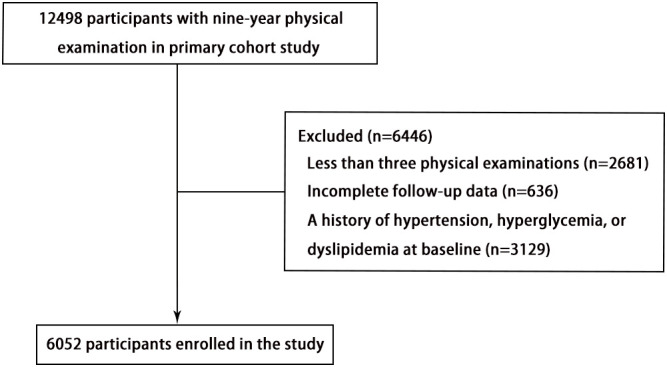
The flowchart illustrating the inclusion process of participants in this study.

This study is a dynamic retrospective cohort study and a secondary analysis of data obtained from Dryad Digital Repository (https://datadryad.org). The study design and characteristics have been previously described (13). Briefly, individuals with local household registration who resided in the community for more than 6 months were eligible for free health check-ups every 2 years based on the China Public Basic Health Services Project. The primary study participants included 12498 adults aged 40 years or older who underwent physical examinations at the Zhanongkou community health service center in Hangzhou, Zhejiang Province, from May 2010 to December 2018. The present study excluded 2681 participants with fewer than three physical examinations, 636 participants without complete data on serum uric acid (SUA), systolic blood pressure (SBP), diastolic blood pressure (DBP), fasting blood glucose (FBG), or total cholesterol (TC), and 3129 participants with a history of hypertension, hyperglycemia, or dyslipidemia at baseline. Ultimately, a total of 6052 individuals (2012 men and 4040 women) were included in this analysis (**Figure 1**).

### 2.2 Data measurement

All participants in the present study underwent baseline examination and at least 2 follow-up examinations. As described in the previous study, trained nurses administered a standardized questionnaire to collect information on demographic characteristics, medical history (including hypertension, diabetes, and dyslipidemia), and medication usage. Weight and height were measured using calibrated scales and a stadiometer, respectively. Blood pressure was measured twice, with a 2-minute interval between measurements, using a calibrated mercury sphygmomanometer. The final value was calculated as the average of the two readings. Venous blood samples were collected from all participants after fasting for at least 8 hours. SUA, fasting blood glucose FBG, total cholesterol TC, and serum creatinine levels were measured using standard clinical laboratory methods.

### 2.3 Data definition

The outcome of this study was the occurrence of hypertension during the follow-up period. Hypertension was defined as systolic blood pressure (SBP) ≥ 140 mmHg or diastolic blood pressure (DBP) ≥ 90 mmHg, self-reported history of hypertension, or current use of antihypertensive medications. Prehypertension was defined as SBP between 120 and 139 mmHg or DBP between 80 and 89 mmHg. HUA was defined as SUA ≥ 416 µmol/L (70mg/L) in men and ≥ 357 µmol/L (60mg/L) in women. The change in SUA was calculated as the highest SUA value during the follow-up period minus the SUA value at baseline, reflecting the control of participants’ SUA levels.

**Figure 2:**
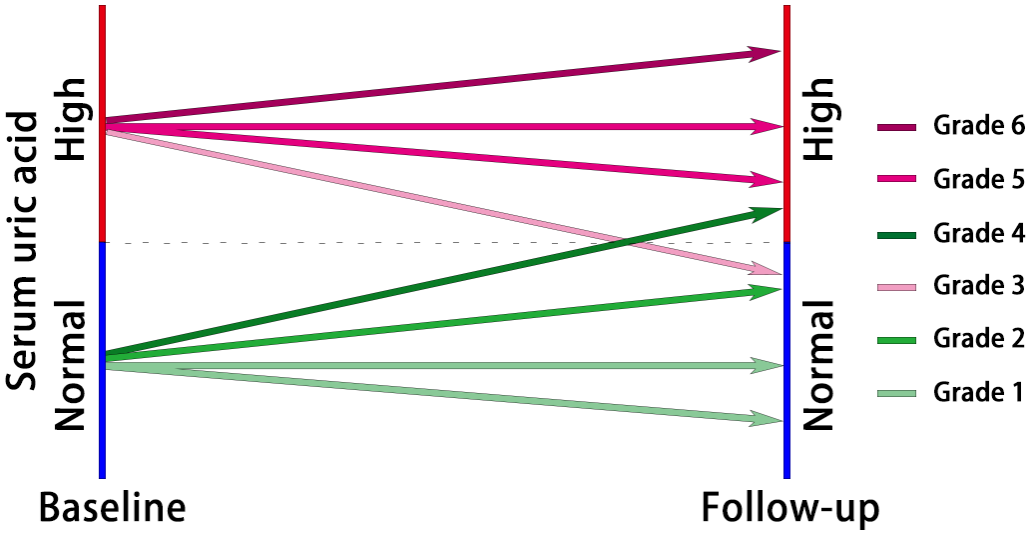
The diagram of participant classification based on grades of serum uric acid control. Grade 1: Normal baseline SUA, consistent or below during follow-up; Grade 2: Normal baseline SUA, increased but within normal ranges during follow-up; Grade 3: Normal baseline SUA, developed HUA during follow-up; Grade 4: HUA at baseline, controlled within normal ranges during follow-up; Grade 5: HUA at baseline, follow-up SUA remained above normal levels but not exceeding baseline; Grade 6: HUA at baseline, follow-up SUA higher than baseline.

The included participants were categorized into six groups based on their baseline SUA levels and the control of SUA levels during the follow-up period (**Figure 2**). Grades 1 to 6 represented the participants’ SUA control from best to worst. Grade 1: Participants had normal SUA levels at baseline, and the highest SUA level during follow-up remained below or consistent with the baseline SUA. Grade 2: Participants had normal baseline SUA levels, and the highest SUA level during follow-up increased but remained within normal ranges. Grade 3: Participants had normal SUA levels at baseline but developed HUA during follow-up. Grade 4: Participants had HUA at baseline, but the highest SUA level during follow-up was controlled within normal ranges. Grade 5: Participants had HUA at baseline, and the highest SUA level during follow-up did not exceed the baseline SUA but remained above normal levels. Grade 6: Participants had HUA at baseline, and the highest SUA level during follow-up was higher than the baseline SUA.

### 2.4 Statistical analysis

Logistic regression models were used to investigate the associations of different classifications of SUA control and hypertension incidence. Model 1 was adjusted for sex and age at baseline. Model 2 was further adjusted for SUA, SBP, and DBP at baseline. Model 3 was further adjusted for body mass index (BMI), FBG, TC, creatinine, and estimated glomerular filtration rate (eGFR) at baseline. *P* values for trend were calculated using the classifications of SUA control grades as a continuous variable in the models. Restricted cubic splines (RCS) were conducted to explore the shape of the relationships between the changes in SUA and hypertension incidence with five knots (at the 5th, 25th, 50th, 75th, and 95th percentiles).

Subgroup analyses and interaction analyses were carried out to discover potential interact impact factors in Model 3. Subgroup analyses were stratified by baseline sex, age (<60 and ≥60 years), BMI (<24 and ≥24 Kg/m^2^), SBP (<120 and ≥120 mmHg), DBP (<80 and ≥80 mmHg), FBG (<6.1 and ≥6.1 mmol/L), TC (<5.18 and ≥5.18 mmol/L), creatinine (<74 and ≥74 μmol/L), and eGFR (<82.1 and ≥82.1 mL/min/1.73m^2^). The cut-off values of creatinine and eGFR were based on their respective baseline medians. The interaction models were established by adding interaction variables to Model 3 and was compared with Model 3 using likelihood ratio test to assess the *P* values for interaction.

Additionally, to verify the robustness of our main results, sensitivity analysis was performed by excluding participants with prehypertension at baseline (n=4492). Another sensitivity analysis was performed by excluding individuals who developed hypertension at the first follow-up examination (n=1154).

Continuous variables are presented as mean ± standard deviation (SD) and categorical variables are presented as frequencies (percentages). Kruskal–Wallis test and Chi-square test were used to inspect the significance of differences in continuous and categorical variables, respectively, between different groups.

All analyses were conducted using SPSS 24.0 software (SPSS Inc., Chicago, IL) and R software version 4.0.5. A two-sided *P* < 0.05 was considered statistically significant.

## 3 Results

### 3.1 Baseline characteristic

The baseline characteristics of the participants are summarized in **Table 1**. The study comprised 6052 participants with an average age of 65.8 ± 9.4 years, among whom 33.2% were men. When compared to participants in grade 1, those with poorer control of SUA tended to be men, older, and exhibit higher baseline BMI, SBP, and DBP.

**Table 1:** Baseline characteristics according to the grades of serum uric acid control.

Characteristics	Overall	Serum uric acid control grades						*P* value
		Grade 1	Grade 2	Grade 3	Grade 4	Grade 5	Grade 6	
No. of participants	6052	964	3183	194	1034	220	457	
Men, n (%)	2012 (33.2)	298 (30.9)	950 (29.8)	77 (39.7)	396 (38.3)	93 (42.3)	198 (43.3)	<0.001
Age, years	65.8 ± 9.4	65.4 ± 9.4	64.3 ± 9.1	68.3 ± 9.2	67.4 ± 9.2	70.0 ± 9.0	70.1 ± 9.6	<0.001
BMI, kg/m^2^	23.2 ± 3.1	22.7 ± 3.0	22.8 ± 3.0	23.8 ± 2.9	24.0 ± 3.0	24.3 ± 3.0	24.9 ± 3.1	<0.001
SBP, mmHg	123.8 ± 10.6	122.7 ± 11.3	122.7 ± 10.7	126.6 ± 9.3	125.8 ± 9.6	126.5 ± 9.4	127.2 ± 9.2	<0.001
DBP, mmHg	75.3 ± 7.1	74.5 ± 7.3	75.1 ± 7.2	75.5 ± 7.1	76.2 ± 6.7	76.1 ± 6.7	76.2 ± 6.9	<0.001
FBG, mmol/L	5.3 ± 0.6	5.3 ± 0.6	5.3 ± 0.6	5.5 ± 0.6	5.3 ± 0.6	5.5 ± 0.6	5.5 ± 0.6	<0.001
TC, mmol/L	4.7 ± 0.8	4.8 ± 0.7	4.7 ± 0.7	4.9 ± 0.7	4.7 ± 0.8	4.8 ± 0.8	4.8 ± 0.8	<0.001
Serum creatinine, μmol/L	74.9 ± 15.6	75.0 ± 15.9	74.0 ± 14.9)	77.4 ± 18.5	75.7 ± 15.4	77.1 ± 17.5	77.2 ± 18.3	<0.001
eGFR, mL/min/1.73m^2^	87.5 ± 26.9	88.4 ± 27.0	91.1 ± 27.2	75.2 ± 24.2	84.8 ± 25.4	76.0 ± 23.7	77.6 ± 24.5	<0.001
SUA, μmol/L	304.7 ± 79.8	304.2 ± 55.3	261.9 ± 53.6	416.6 ± 45.9	327.4 ± 50.8	456.4 ± 55.6	432.3 ± 55.1	<0.001

BMI, body mass index; SBP, systolic blood pressure; DBP, diastolic blood pressure; FBG, fasting blood glucose; TC, total cholesterol; eGFR, estimated glomerular filtration rate; SUA, serum uric acid.

### 3.2 Association between hypertension incidence and the control of SUA

During a median follow-up period of 6 years, 2550 (42.1%) participants developed hypertension. The relationship between the risk of hypertension and the grades of SUA control is presented in **Table 2**. The incidence of hypertension gradually increased with poorer SUA control, rising from 33.8% in grade 1 to 60.6% in grade 6. Comparing to grade 1 of SUA control, the odds ratios (ORs) for hypertension incidence were 1.216 (1.045-1.415) in grade 2, 1.659 (1.214-2.267) in grade 3, 1.965 (1.640-2.354) in grade 4, 2.348 (1.745-3.161) in grade 5, and 3.012 (2.392-3.791) in grade 6. Similar patterns were observed in Model 1, adjusted for sex and age. After further adjustment for additional potential confounding factors in Model 2, the OR for hypertension incidence was 1.354 (1.116-1.644) in grade 2 of SUA control, surpassing the OR of 1.147 (0.771-1.707) in grade 3. Consistent findings were demonstrated in Model 3, accounting for all potential confounding factors. Participants with poorer SUA control were significantly more likely to develop hypertension (*P* for trend < 0.001).

**Table 2:** ORs for risk of hypertension development according to the grades of serum uric acid control.

		Crude	Model 1	Model 2	Model 3
	Case, n (%)	OR (95%CI)	OR (95%CI)	OR (95%CI)	OR (95%CI)
Total	2550 (42.1)				
Grade 1	326 (33.8)	Reference	Reference	Reference	Reference
Grade 2	1220 (38.3)	1.216 (1.045-1.415)	1.288 (1.104-1.504)	1.354 (1.116-1.644)	1.347 (1.109-1.636)
Grade 3	89 (45.9)	1.659 (1.214-2.267)	1.492 (1.085-2.051)	1.147 (0.771-1.707)	1.138 (0.764-1.693)
Grade 4	518 (50.1)	1.965 (1.640-2.354)	1.849 (1.538-2.223)	1.573 (1.264-1.958)	1.552 (1.245-1.934)
Grade 5	120 (54.5)	2.348 (1.745-3.161)	1.988 (1.468-2.691)	1.781 (1.182-2.684)	1.765 (1.170-2.663)
Grade 6	277 (60.6)	3.012 (2.392-3.791)	2.559 (2.023-3.238)	2.218 (1.607-3.061)	2.165 (1.566-2.993)
*P* for trend		<0.001	<0.001	<0.001	<0.001

OR, odds ratio; CI, confidence interval.

Model 1: adjusted for age and sex at baseline.

Model 2: adjusted for variables in Model 1 plus SBP, DBP and SUA at baseline.

Model 3: adjusted for variables in Model 2 plus BMI, FBG, TC, serum creatinine and eGFR at baseline.

**Figure 3:**
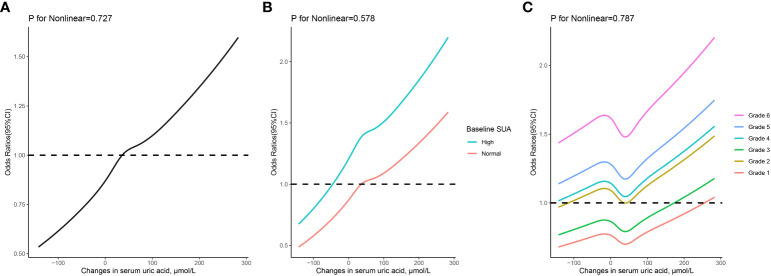
The relationship between changes in serum uric acid and risk of hypertension based on restricted cubic spines with 5 knots at 5th, 25th, 50th, 75th, and 95th percentiles. **(A)** all participants; **(B)** participants classified based on baseline serum uric acid levels; **(C)** participants classified based on grades of serum uric acid control. Adjusted for sex, age, body mass index, serum uric acid, systolic and diastolic blood pressure, fasting blood glucose, total cholesterol, serum creatinine, and estimated glomerular filtration rate at baseline.

As depicted in **Figure 3**, the RCS regression models demonstrated a linear correlation between the risk of hypertension and the dynamic changes in SUA. When classified based on baseline SUA levels, participants with HUA at baseline exhibited a higher risk of hypertension events compared to those with normal baseline SUA, even when their dynamic changes in SUA were the same. Additionally, linear relationships between dynamic changes in SUA across different grades of SUA control and the risk of hypertension were observed.

### 3.3 Subgroup analyses

The results of subgroup analyses are summarized in **Table 3**. Generally, participants with poorer SUA control, particularly those in grade 6, exhibited a significantly higher risk of developing hypertension within each subgroup. Significant interactions were observed in both SBP (*P* for interaction = 0.003) and DBP (*P* for interaction < 0.001) subgroups. In these two subgroups, comparing participants with poor SUA control with participants to those with the best SUA control in their respective subgroups, the increased risk of developing hypertension in participants with a baseline SBP <120 mmHg or DBP <80 mmHg was obviously higher than that in participants with a baseline SBP ≥120 mmHg or DBP ≥80mmHg.

**Table 3:** Subgroup analyses for the risk of hypertension by the grades of serum uric acid control.

Variables, Case/Total	Serum uric acid control						*P* for interaction
	Grade 1	Grade 2	Grade 3	Grade 4	Grade 5	Grade 6	
Sex Male, 965/2012 Female, 1585/4040	Reference Reference	1.611 (1.157-2.245) 1.222 (0.959-1.556)	0.869 (0.458-1.647) 1.357 (0.815-2.260)	1.654 (1.154-2.370) 1.506 (1.139-1.993)	0.946 (0.499-1.794) 2.839 (1.642-4.909)	2.276 (1.359-3.811) 2.042 (1.341-3.108)	0.075
Age, year <60, 413/1505 ≥60, 2137/4547	Reference Reference	1.260 (0.837-1.896) 1.403 (1.122-1.755)	1.913 (0.730-5.017) 1.032 (0.661-1.611)	1.881 (1.161-3.048) 1.499 (1.168-1.924)	5.181 (1.836-14.620) 1.472 (0.934-2.321)	2.223 (0.959-5.155) 2.147 (1.495-3.083)	0.250
BMI, Kg/m^2^ <24, 1406/3776 ≥24, 1144/2276	Reference Reference	1.350 (1.059-1.721) 1.371 (0.988-1.902)	1.091 (0.639-1.862) 1.203 (0.660-2.192)	1.714 (1.278-2.299) 1.414 (1.006-1.986)	1.446 (0.810-2.580) 2.193 (1.209-3.977)	2.186 (1.375-3.477) 2.183 (1.368-3.482)	0.732
SBP, mmHg <120, 187/1814 ≥120, 2363/4238	Reference Reference	1.488 (0.872-2.539) 1.349 (1.086-1.676)	3.845 (1.395-10.592) 0.938 (0.606-1.451)	2.544 (1.395-4.642) 1.442 (1.131-1.840)	3.743 (1.386-10.109) 1.489 (0.945-2.347)	2.974 (1.222-7.235) 2.089 (1.460-2.988)	0.003
DBP, mmHg <80, 1335/4017 ≥80, 1215/2035	Reference Reference	1.260 (0.984-1.614) 1.424 (1.032-1.967)	1.707 (1.040-2.804) 0.532 (0.276-1.026)	1.668 (1.261-2.208) 1.368 (0.951-1.968)	2.834 (1.674-4.797) 0.801 (0.414-1.551)	2.279 (1.513-3.431) 2.006 (1.152-3.494)	<0.001
FBG, mmol/L <6.1, 2205/5379 ≥6.1, 345/673	Reference Reference	1.283 (1.043-1.579) 1.923 (1.087-3.402)	1.183 (0.763-1.835) 0.871 (0.320-2.368)	1.561 (1.234-1.974) 1.390 (0.729-2.649)	1.666 (1.056-2.627) 1.864 (0.656-5.295)	2.253 (1.584-3.205) 1.639 (0.692-3.887)	0.218
TC, mmol/L <5.18, 1787/4039 ≥5.18, 763/1743	Reference Reference	1.427 (1.132-1.799) 1.167 (0.812-1.676)	1.236 (0.757-2.018) 0.945 (0.470-1.903)	1.620 (1.246-2.107) 1.390 (0.924-2.092)	1.739 (1.047-2.890) 1.841 (0.894-3.792)	2.072 (1.397-3.072) 2.361 (1.313-4.245)	0.931
Creatinine, μmol/L <74, 1175/2866 ≥74, 1375/3186	Reference Reference	1.341 (1.017-1.767) 1.368 (1.037-1.803)	0.911 (0.512-1.619) 1.463 (0.833-2.569)	1.726 (1.256-2.372) 1.448 (1.064-1.971)	1.550 (0.850-2.826) 1.924 (1.088-3.403)	2.706 (1.681-4.354) 1.830 (1.171-2.859)	0.252
eGFR, mL/min/1.73m^2^ <82.1, 1405/3024 ≥82.1, 1145/3028	Reference Reference	1.551 (1.153-2.087) 1.259 (0.967-1.638)	0.694 (0.400-1.205) 1.899 (1.020-3.538)	1.542 (1.117-2.127) 1.607 (1.177-2.194)	1.495 (0.837-2.673) 1.787 (0.968-3.299)	2.009 (1.257-3.211) 2.340 (1.464-3.742)	0.190

BMI, body mass index; SBP, systolic blood pressure; DBP, diastolic blood pressure; FBG, fasting blood glucose; TC, total cholesterol; eGFR, estimated glomerular filtration rate.

### 3.4 Sensitivity analyses

The results of sensitivity analyses are presented in **Table 4**. After excluding participants with prehypertension at baseline, those with poorer SUA control (grades 2 to 6) exhibited an elevated risk of hypertension incidence in comparison to those with optimal SUA control (grade 1). Similar findings were observed after excluding participants who developed hypertension at the first follow-up.

**Table 4:** Sensitivity analyses for risk of hypertension incidence.

	Sensitivity analysis 1		Sensitivity analysis 2	
	Case/Total	OR (95%CI)	Case/Total	OR (95%CI)
Total	136/1560		1396/4898	
Grade 1	14/296	Reference	174/812	Reference
Grade 2	64/922	1.225 (0.658-2.278)	683/2646	1.446 (1.147-1.822)
Grade 3	8/35	8.988 (2.965-27.244)	45/150	1.050 (0.652-1.692)
Grade 4	29/199	3.183 (1.598-6.341)	288/804	1.582 (1.221-2.050)
Grade 5	10/41	9.596 (3.121-29.507)	55/155	1.504 (0.925-2.444)
Grade 6	11/66	5.326 (1.947-14.573)	151/331	2.019 (1.389-2.933)
*P* for trend		<0.001		<0.001

OR, odds ratio; CI, confidence interval.

Sensitivity analysis 1: adjusted for variables in Model 3 after excluding participants with prehypertension at baseline.

Sensitivity analysis 2: adjusted for variables in Model 3 after excluding participants with hypertension development at the first follow-up.

## 4 Discussion

In this retrospective cohort study, we explored the relationship between the incidence of hypertension and the grades of SUA control. Our findings highlight a substantial elevation in the risk of developing hypertension among individuals with poor SUA control, irrespective of their baseline SUA levels. In addition, our results indicated that individuals presenting HUA at baseline faced a heightened risk of hypertension compared to those with normal baseline SUA levels when experiencing the same changes in SUA.

Recently, numerous studies have revealed the association between SUA and hypertension. A growing body of evidence suggests that SUA serves as an independent risk factor for the onset and progression of hypertension (10–12). In a meta-analysis encompassing 25 studies with 97,824 participants, the relative risk of incident hypertension was found to increase by 1.15 for every 1 mg/dl rise in SUA (9). Notably, SUA is a dynamic biochemical parameter that undergoes changes over time. A singular measurement of SUA at a specific time point may introduce bias to the relationship, as it fails to consider how SUA change within individual over time. Additionally, neglecting the potential impact of SUA changes and their association with future hypertension risk further compounds this bias. However, limited information is available on the association of change over time in SUA to hypertension incidence and blood pressure progression. Our research serves to supplement and provide new insights into this area. The Pressioni Arteriose Monitorate E loro Associazioni study (14) found that individuals who had a normal baseline SUA and developed HUA during a 25-year follow-up period showed higher 24-hour blood pressure. A prospective cohort study using trajectories to reflect an over 5-year change in SUA demonstrated distinct trajectories of uric acid were differentially associated with hypertension risk in middle-aged adults (15). Another prospective cohort study indicated that an increase in SUA over time could independently predict progression from prehypertension to hypertension, with an odds ratio of 1.41 in the highest quartile versus the lowest quartile (16). Our results were similar with these studies, showing that individuals with poorly controlled SUA faced a significantly higher risk of developing hypertension compared to those with optimally controlled SUA. A prior study with a large Korean cohort similarly indicated elevated risks for incident hypertension in the highest quartiles of SUA change compared to the lowest quartiles, supporting our findings (17).

In contrast to previous studies that assessed hypertension risk based on quartiles of SUA changes, our study adopted a novel practical classification method, categorizing SUA control into six grades. This classification considers whether baseline SUA was high and whether changes in SUA progressed to HUA or decreased to normal levels. To our knowledge, this is the first study to employ such a method to classify changes in SUA. Each category represented a distinct grade of SUA control, offering a more intuitive approach for conveying the risk of hypertension at different SUA change levels to the general population.

Notably, after adjusting for all confounding factors in Model 3, participants with normal baseline SUA levels that increased during follow-up exhibited an elevated risk of developing hypertension, even though their SUA levels remained within the normal range throughout. More interestingly, participants with baseline HUA whose SUA decreased to normal levels displayed a lower risk of incident hypertension compared to those with increased SUA levels during the follow-up period but still remaining within normal ranges. This underscores the critical role of good control of SUA, where a reduction from HUA at baseline to normal levels significantly diminishes the risk of hypertension. Conversely, an increase in SUA levels, even within the normal range, is associated with an elevated risk of hypertension. In addition, the linear associations persisted across various grades of SUA control, also emphasizing the importance of SUA management in the prevention of hypertension. Similar to our results, the Brisighella Heart Study (18) revealed a significant increase in SBP among individuals with worsened SUA levels. However, they simply categorized the population into groups based on uric acid changes: unchanged, elevated, or improved. In our study, when grouping participants, we considered whether their SUA levels deteriorated to hyperuricemia or improved to normal levels. This classification method was more detailed and practical.

Furthermore, an increased risk of hypertension was observed in participants with HUA at baseline compared to those with normal SUA at baseline when their changes in SUA were identical, implying that elevated baseline SUA levels also promoted the onset of hypertension. Thus, both elevated SUA levels at baseline and poor control of SUA can increased the risk of developing hypertension, which is consistent with the previous report (17). A prior study (19) has confirmed that the combined effect between increased baseline and changes in SUA is a significant and independent determinant for metabolic syndrome, providing evidence for our results.

Recently, the Uric Acid Right for Heart Health (URRAH) study proposed a lower SUA cutoff value, which may be more suitable for cardiovascular risk factors and cardiovascular mortality (20, 21). But we did not adopt this cutoff value for two main reasons. Firstly, there was a difference in the racial composition of the study populations. The URRAH study primarily included Italians, while our study focused on Chinese individuals. Different racial groups exhibit variations in SUA metabolism rates and disease incidence (22). Secondly, the observed outcomes of the studies were different. The URRAH study focused on cardiovascular mortality, while our study investigated the incidence of hypertension. Therefore, in our study, we conducted the analyses with the classical hyperuricemia cutoff value, which is widely used and accepted in China.

Subgroup analyses indicated that there was an interaction between SBP, DBP and grades of SUA control in the development of hypertension. When SBP <120 mmHg or DBP <80 mmHg, the risk of developing hypertension due to poor SUA control is higher. After excluding participants in prehypertension at baseline or participants who developed hypertension at the first follow-up, the sensitivity analyses further confirmed that the control of SUA was closely related to the risk of hypertension even though the blood pressure was at an ideal level. Participants with the best control had the lowest risk. These results are a reminder of the need to keep SUA under control even when blood pressure is normal.

The potential mechanisms of SUA contributes to the development of hypertension are multifaceted and complex. First, uric acid causes hypertension in the rat via activating the renin-angiotensin system inducing vascular smooth muscle cell proliferation, oxidative stress, and renal arteriolopathy (23–25). Second, HUA can induce endothelial dysfunction and renal vasoconstriction by inhibiting Nitric Oxide production. These factors cause renal structure abnormalities, resulting in salt-sensitive and uric acid-independent hypertension (26, 27). Third, the formation of uric acid is accompanied by the production of reactive oxygen species (28). Oxidative stress induced by reactive oxygen species can causes vascular dysfunction, cardiovascular remodeling, and renal dysfunction, leading to hypertension (29). In addition, many studies have suggested that uric acid–lowering medication can decrease blood pressure (30–32). Therefore, SUA is closely related to the development of hypertension.

The advantages of this study included a large sample size, a long follow-up period, and multiple follow-up measurements of SUA levels and blood pressure. Considering the impact of dynamic changes in SUA on the onset of hypertension, our results would be more reliable. The present study also has several limitations that need to be noted. First of all, this was a single-center study involving participants older than 40 years. Thus, the data predominantly reflect a specific demographic within a particular region, which might limit the generalizability of our findings to broader populations. Second, although we accounted for several confounding factors, certain potential factors influencing the development of hypertension, such as family history of hypertension, drinking and smoking, were not included in our analysis. Third, the results might be biased, because our study was a retrospective analysis. Finally, during the follow-up, some individuals who developed hypertension may have used diuretics and losartan to control blood pressure, and these medications can affect SUA levels (33, 34). However, information on these medication uses was not available, which could potentially impact our results.

## 5 Conclusion

In conclusion, poor SUA control increases risk for developing hypertension. Regardless of whether the initial SUA is normal or not, an increase in SUA over time rises the risk of developing hypertension. On this basis, initial high uric acid will further exacerbate this risk. Therefore, monitoring changes in SUA and implementing effective SUA control are important measures for primary prevention of hypertension.

## Data availability statement

Publicly available datasets were analyzed in this study. This data can be found here: https://doi.org/10.5061/dryad.z08kprrk1.

## Ethics statement

The original study, the source of our research data, involving humans was approved by Ethics Committee of Hangzhou Normal University School of Public Health (20220009). The protocol of this study was approved by the Ethics Committee of the Second Affiliated Hospital of Shantou University Medical College. No informed consent was required because the study was conducted retrospectively and anonymized.

## Author contributions

ZYL: Conceptualization, Writing – original draft, Writing – review & editing. SW: Data curation, Methodology, Writing – review & editing. ZC: Investigation, Writing – review & editing. WL: Investigation, Writing – review & editing. ZHL: Data curation, Writing – review & editing. HS: Data curation, Writing – review & editing. DG: Conceptualization, Methodology, Supervision, Writing – review & editing.
